# Myocardial Injury After Ischemia/Reperfusion Is Attenuated By Pharmacological Galectin-3 Inhibition

**DOI:** 10.1038/s41598-019-46119-6

**Published:** 2019-07-03

**Authors:** Jaime Ibarrola, Lara Matilla, Ernesto Martínez-Martínez, Alexandre Gueret, Amaya Fernández-Celis, Jean-Paul Henry, Lionel Nicol, Frederic Jaisser, Paul Mulder, Antoine Ouvrard-Pascaud, Natalia López-Andrés

**Affiliations:** 1 Cardiovascular Translational Research. Navarrabiomed, Complejo Hospitalario de Navarra (CHN), Universidad Pública de Navarra (UPNA), IdiSNA, Pamplona, Spain; 2 Normandie University UNIROUEN, Rouen, France; 3 INSERM, UMR1096 Rouen, France; 4 Inserm 1138, Institut des Cordeliers, Paris, France; 5 Université de Lorraine, INSERM, Centre d’Investigations Cliniques-Plurithématique 1433, UMR 1116, CHRU de Nancy, Nancy, France

**Keywords:** Heart failure, Molecular biology

## Abstract

Although optimal therapy for myocardial infarction includes reperfusion to restore blood flow to the ischemic region, ischemia/reperfusion (IR) also initiates an inflammatory response likely contributing to adverse left ventricular (LV) extracellular matrix (ECM) remodeling. Galectin-3 (Gal-3), a β-galactoside-binding-lectin, promotes cardiac remodeling and dysfunction. Our aim is to investigate whether Gal-3 pharmacological inhibition using modified citrus pectin (MCP) improves cardiac remodeling and functional changes associated with IR. Wistar rats were treated with MCP from 1 day before until 8 days after IR (coronary artery ligation) injury. Invasive hemodynamics revealed that both LV contractility and LV compliance were impaired in IR rats. LV compliance was improved by MCP treatment 8 days after IR. Cardiac magnetic resonance imaging showed decreased LV perfusion in IR rats, which was improved with MCP. There was no difference in LV hypertrophy in MCP-treated compared to untreated IR rats. However, MCP treatment decreased the ischemic area as well as Gal-3 expression. Gal-3 blockade paralleled lower myocardial inflammation and reduced fibrosis. These novel data showing the benefits of MCP in compliance and ECM remodeling in IR reinforces previously published data showing the therapeutic potential of Gal-3 inhibition.

## Introduction

The primary pathological expression of coronary artery disease is myocardial injury resulting from an ischemia/reperfusion (IR) insult. Myocardial IR injury is a complex event with many interlinked processes, and it is a strong inductor of adverse left ventricular (LV) extracellular matrix (ECM) remodeling^1^. Abnormal ECM turnover results in activation of cellular and molecular events that lead to structural instability. Moreover, IR activates an inflammatory response where inflammatory cells are recruited to the ischemic area, exacerbating cardiomyocyte death^2^. Understanding ECM and inflammatory changes is important for identifying factors that facilitates the adverse LV remodeling that leads to progressive ventricular dysfunction and congestive heart failure (HF). Importantly, approaches to combat this phenomenon are not adequately developed, and it is necessary to find out an agent that reduces myocardial IR injury and minimize the damage.

Galectin-3 (Gal-3) is a 29 to 35 kDa protein, member of a β-galactoside binding lectin family expressed in cardiac cells which has emerged as a potential regulator of physiological and pathological processes including inflammation and fibrosis^3^. Modified citrus pectin (MCP) (a complex water soluble indigestible polysaccharide riche in β-galactose) is a Gal-3 inhibitor that blocks the lectin’s activity^4^. We and others have demonstrated that Gal-3 inhibition improves cardiovascular remodeling in several pathological conditions such as hyperaldosteronism, obesity, hypertension, angiotensin II treatment, pressure overload and aortic stenosis^5–8^. Based on our previous findings demonstrating the cardioprotective efficacy of the Gal-3 inhibitor MCP in a variety of cardiovascular pathologies^5,6,8,9^, we tested the hypothesis that MCP treatment provides cardioprotection against LV remodeling associated to IR injury in rats, delaying the adverse remodeling that leads to progressive LV dysfunction and HF. To that purpose, we evaluated the early benefits of Gal-3 inhibition on cardiac functional, histological and molecular alterations in the rat model of IR.

## Results

### Effects of Gal-3 inhibition on cardiac function and myocardial perfusion in IR rats

**Figure 1:**
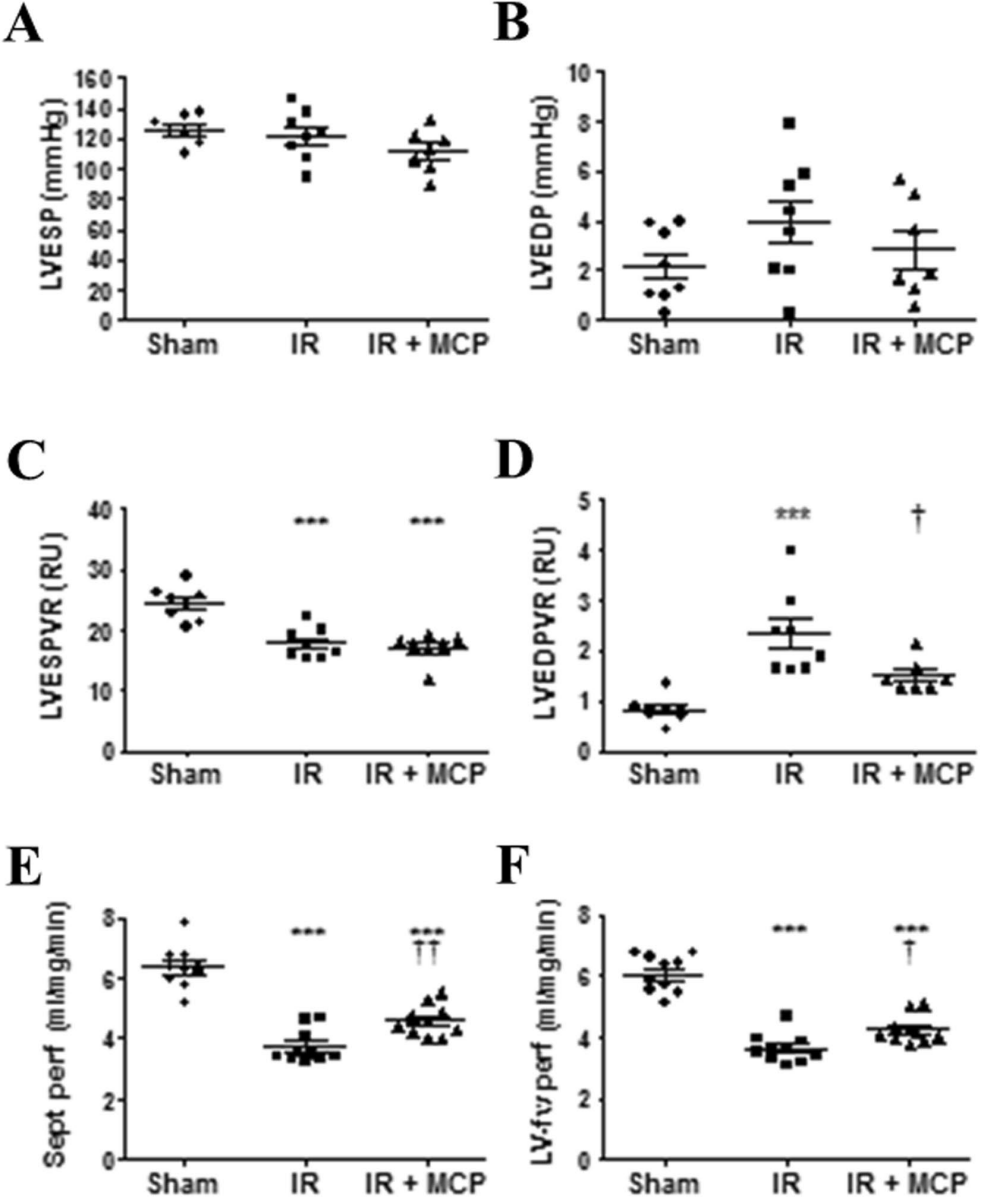
LV hemodynamics and perfusion measurements. (**A,B**) LV end-systolic pressure (LVESP) and LV end-diastolic pressure (LVEDP) in Sham-operated (circle, n = 8), after ischemia-reperfusion (IR) (square, n = 8) and in MCP-treated IR rats (triangle, n = 7). (**C,D**) LV end-systolic pressure-volume relation (LVESPVR i.e. LV contractility) and LV end-diastolic pressure-volume relation (LVEDPVR i.e. LV compliance) in Sham-operated (circle, n = 8), after ischemia-reperfusion (IR) (square, n = 8) and in MCP-treated IR rats (triangle, n = 7). (**E,F**) Interventricular septum (Sept) and LV-free wall (LV-fw) myocardial perfusion in Sham-operated (circle, n = 10), after ischemia-reperfusion (IR) (square, n = 10) and in MCP-treated IR rats (triangle, n = 10). Histogram bars represent the mean ± SEM of each group. ***p < 0.001 vs. Sham-operated; ^†^p < 0.05 and ^††^p < 0.01 vs. IR.

Magnetic resonance imaging (MRI) measurements showed that IR-associated injuries are responsible for increased LV end-systolic and LV end-diastolic volumes (LVESV and LVEDV), likely contributing to reductions in LV ejection fraction and in cardiac output (Table 1). Invasive hemodynamics indicated that the load-dependent rates dP/dt_max_ and dP/dt_min_ of LV pressure rise and fall, taken as indexes of LV global systolic contractility and diastolic relaxation respectively, were altered after IR (Table 1). Neither LV end-systolic pressure (LVESP) nor LV end-diastolic pressure (LVEDP) was modified (Fig. 1A,B). However, LV pressure-volume curves that allow the load-independent assessments of LV systolic contractility and of LV compliance in diastole, via the calculation of the LV end-systolic or end-diastolic pressure-volume relations respectively (LVESPVR and LVEDPVR), indicated that both were altered at 8 days post-IR (Fig. 1C,D). Nevertheless, in MCP treated compared to untreated IR rats, a trend toward the improvement of the LV filling pressure i.e. LV end-diastolic pressure (LVEDP) (Fig. 1A,B) paralleled clear amelioration of the calculated LV compliance in diastole (LVEDPVR; Fig. 1C,D). In the same way, the relaxation constant τau_1/2_ was unaltered in MCP treated IR rats compared to Sham-operated (Table 1). Concerning myocardial perfusion which takes place during the diastolic phase of the cardiac cycle, MRI measurements indicated that IR induced a decrease in LV perfusion, as evidenced in both the interventricular septum and the LV free wall (Fig. 1E,F). Interestingly, 8 days of MCP treatment partly improved myocardial perfusion in comparison to untreated IR rats (Fig. 1E,F).

**Table 1:** Effect of the inhibition of Gal-3 on cardiac parameters in IR rats.

	Sham	IR	IR + MCP
**Weights**	n = 10	n = 12	n = 10
Body (g)	323 ± 5	297 ± 7*	294 ± 8*
Heart (mg)	1036 ± 22	974 ± 27	992 ± 31
LV (mg)	719 ± 17	691 ± 21	698 ± 22
HW/BW (mg/g)	3.21 ± 0.06	3.29 ± 0.09	3.38 ± 0.06
LVW/BW (mg/g)	2.23 ± 0.05	2.33 ± 0.06	2.38 ± 0.05
**Function (MRI)**	n = 10	n = 9	n = 9
HR (bpm)	403 ± 11	349 ± 12*	343 ± 18*
LVESV (μl)	102 ± 5	190 ± 15***	224 ± 22***
LVEDV (μl)	352 ± 14	433 ± 22*	466 ± 32**
EF (%)	71 ± 1	57 ± 2***	52 ± 2***
SV (µl)	250 ± 10	244 ± 12	242 ± 17
CO (ml/min)	100 ± 3	84 ± 4*	83 ± 6*
**Perfusion (MRI)**	n = 10	n = 10	n = 10
LV perf (ml/mg/min)	6.09 ± 0.19	3.64 ± 0.15***	4.27 ± 0.14***^,†^
Septum perf (ml/mg/min)	6.35 ± 0.22	3.75 ± 0.17***	4.60 ± 0.17***^,††^
**Hemodynamics**	n = 8	n = 8	n = 7
HR (bpm)	417 ± 14	394 ± 14	379 ± 13
SBP (mmHg)	132 ± 5	124 ± 5	121 ± 3
DBP (mmHg)	94 ± 4	92 ± 5	90 ± 3
LVESP	125.7 ± 3.2	121.9 ± 5.9	111.9 ± 5.3
LVEDP	2.2 ± 0.5	4.0 ± 0.9	2.9 ± 0.7
dP/dt max	9784 ± 294	8283 ± 452	7968 ± 572*
dP/dt min	8504 ± 392	6889 ± 362*	6591 ± 321**
LVESPVR	24.38 ± 0.98	17.83 ± 0.90***	17.19 ± 0.91***
LVEDPVR	0.82 ± 0.09	2.32 ± 0.30***	1.51 ± 0.12^†^
Tau e	5.34 ± 0.20	7.63 ± 0.32***	6.71 ± 0.43*
Tau 1/2	4.19 ± 0.18	5.29 ± 0.38*	4.44 ± 0.22
**Serum parameters**	n = 10	n = 10	n = 10
BNP (AU)	0.16 ± 0.014	0.26 ± 0.024*	0.19 ± 0.024^†^
IL-1 β (AU)	127 ± 14	184 ± 12*	139 ± 11^†^
CRP (AU)	2667 ± 410	4231 ± 425*	2436 ± 235^†^
**Hemodynamics**	n = 8	n = 8	n = 7
HR (bpm)	417 ± 14	394 ± 14	379 ± 13
SBP (mmHg)	132 ± 5	124 ± 5	121 ± 3
DBP (mmHg)	94 ± 4	92 ± 5	90 ± 3
LVESP	125.7 ± 3.2	121.9 ± 5.9	111.9 ± 5.3
LVEDP	2.2 ± 0.5	4.0 ± 0.9	2.9 ± 0.7
dP/dt max	9784 ± 294	8283 ± 452	7968 ± 572*
dP/dt min	8504 ± 392	6889 ± 362*	6591 ± 321**
LVESPVR	24.38 ± 0.98	17.83 ± 0.90***	17.19 ± 0.91***
LVEDPVR	0.82 ± 0.09	2.32 ± 0.30***	1.51 ± 0.12^†^
Tau e	5.34 ± 0.20	7.63 ± 0.32***	6.71 ± 0.43*
Tau 1/2	4.19 ± 0.18	5.29 ± 0.38*	4.44 ± 0.22
**Serum parameters**	n = 10	n = 10	n = 10
BNP (AU)	0.16 ± 0.014	0.26 ± 0.024*	0.19 ± 0.024^†^
IL-1 β (AU)	127 ± 14	184 ± 12*	139 ± 11†
CRP (AU)	2667 ± 410	4231 ± 425*	2436 ± 235^†^

IR: ischemia reperfusion; MCP: modified citrus pectin; LV: Left ventricle; LVW: LV weight; BW: Body weight; HW: heart weight; HR: heart rate; LVESV: LV end-systolic volume; LVEDV: LV end-diastolic volume; EF: ejection fraction; SV: stroke volume; CO: cardiac output; SBP: systolic blood pressure; DBP: diastolic blood pressure; LVESP: LV end-systolic pressure; LVEDP: LV end-diastolic pressure; LVESPVR: LV end-systolic pressure-volume relation; LVEDPVR: LV end-diastolic pressure-volume relation; BNP: bran natriuretic peptide; IL-1 β: interleukin-1 beta; CRP: C-reactive protein. Data values represent mean ± SEM number of rats per group is indicated in each column. *p < 0.05, **p < 0.01 *** p < 0.001 *vs*. control group; ^†^p < 0.05 *vs*. IR group. Anova plus Tukey for weight and functional measurements and plus Bonferroni for protein quantification.

Serum brain natriuretic peptide (BNP), an early marker predicting the structural and/or functional heart changes, was increased (p < 0.05) in rats with IR but normalized by Gal-3 blockade with MCP treatment (Table 1), showing the need to further study LV remodeling.

MCP treatment did not affect all measured parameters in the absence of IR.

### Effects of Gal-3 blockade on cardiac structure and Gal-3 expression

**Figure 2:**
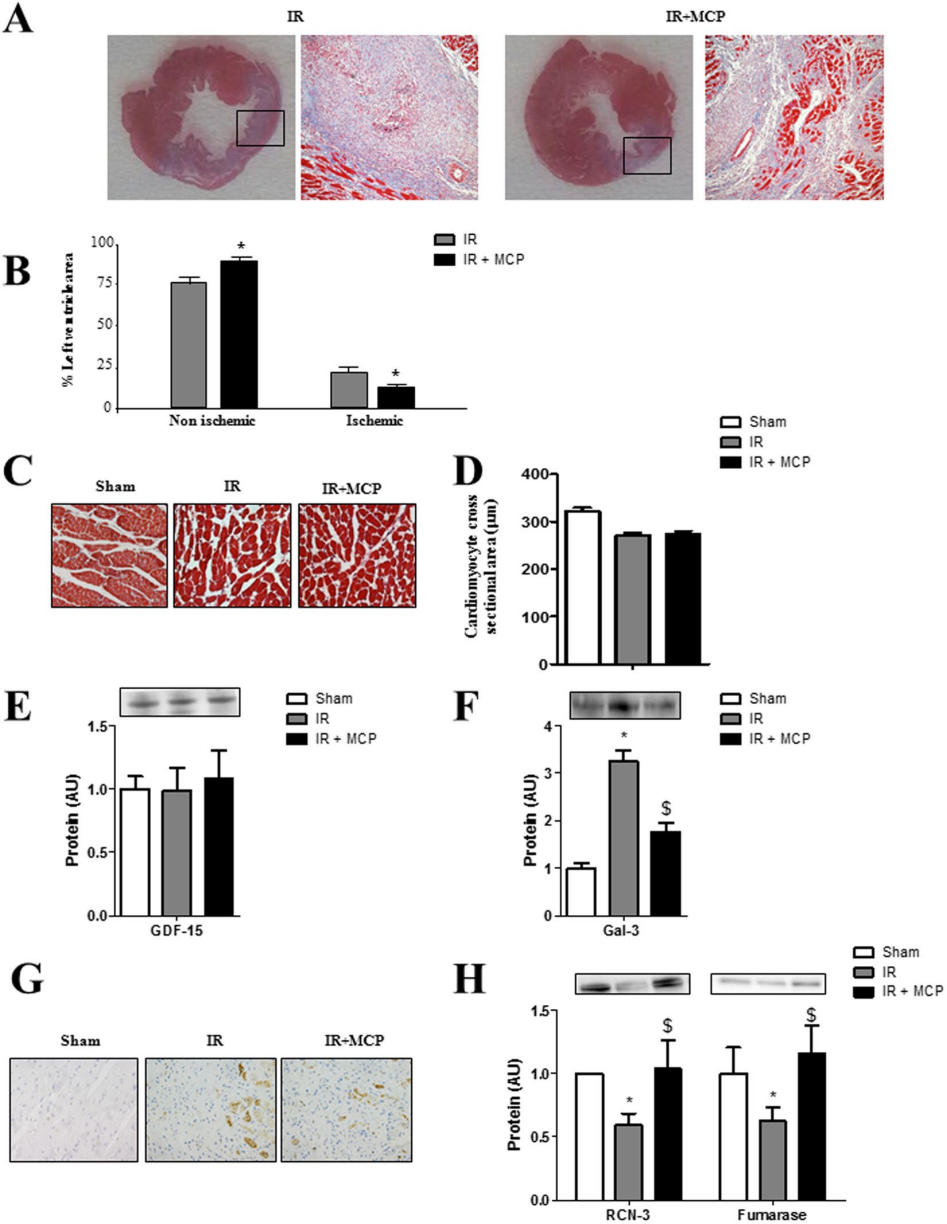
Myocardial cross sections of IR and IR + MCP-treated rats and quantification of Gal-3 expression. (**A**) Representative microphotographs of myocardial sections were stained with Masson trichrome and imaged at 4x and 40x power fields. (**B**) Ischemic areas were selected and quantified using computed-based image analysis software. (**C,D**) Cardiomyocyte cross sectional area was measured in myocardial sections. (**E**) Quantification of GDF-15 protein expression. (**F**) Quantification of Gal-3 protein levels in sham, IR and IR + MCP rats. (**G**) Representative microphotographs imaged at 40x of the ischemic zone stained for Gal-3. (**H**) Quantification of Gal-3 downstream proteins RCN-3 and fumarase. Histogram bars represent the mean ± SEM of each group of animals (n ≥ 12 per group) in arbitrary units or as a percentage of staining normalized to HPRT, 18 s and β-actin or stain-free gel for cDNA and protein respectively. *p < 0.01 *vs*. Sham, $, p < 0.01 *vs*.IR.

Representative LV microphotographs of IR rats treated or not with the Gal-3 inhibitor MCP shown in Fig. 2A were used to measure LV ischemic and non ischemic areas. Treatment allowed increasing the non-ischemic LV area and decreasing the LV ischemic area (Fig. 2B).

There was no difference neither in LV weight (Table 1) nor in cardiomyocyte mean cross sectional area (Fig. 2C,D) at this early stage of the disease. This was confirmed by the absence of changes in GDF-15 (growth differentiation factor-15) protein expression, a cytokine whose increased expression has been involved in induction of cardiomyocyte hypertrophy^10^ (Fig. 2E). Interestingly, cardiac Gal-3 protein expression was increased (p < 0.01) in IR animals and it was reduced (p < 0.01) in the ischemic zone of MCP-treated IR rats (Fig. 2F). Detection of Gal-3 by immunohistochemistry confirmed its increased expression in the myocardium of IR rats as compared to controls and its lowered expression in the myocardium of MCP-treated animals as compared to IR untreated rats (Fig. 2G). In order to confirm that MCP was blocking Gal-3 downstream proteins, reticulocalbin-3 (RCN-3) and fumarase two proteins recently described to be down-regulated by Gal-3 were analyzed^11,12^. The levels of protein expression of both RCN-3 and fumarase were decreased in myocardium from IR rats and restored by MCP treatment (Fig. 2H). MCP treatment did not affect all measured parameters in the absence of IR.

### Effects of Gal-3 blockade on cardiac inflammation

**Figure 3:**
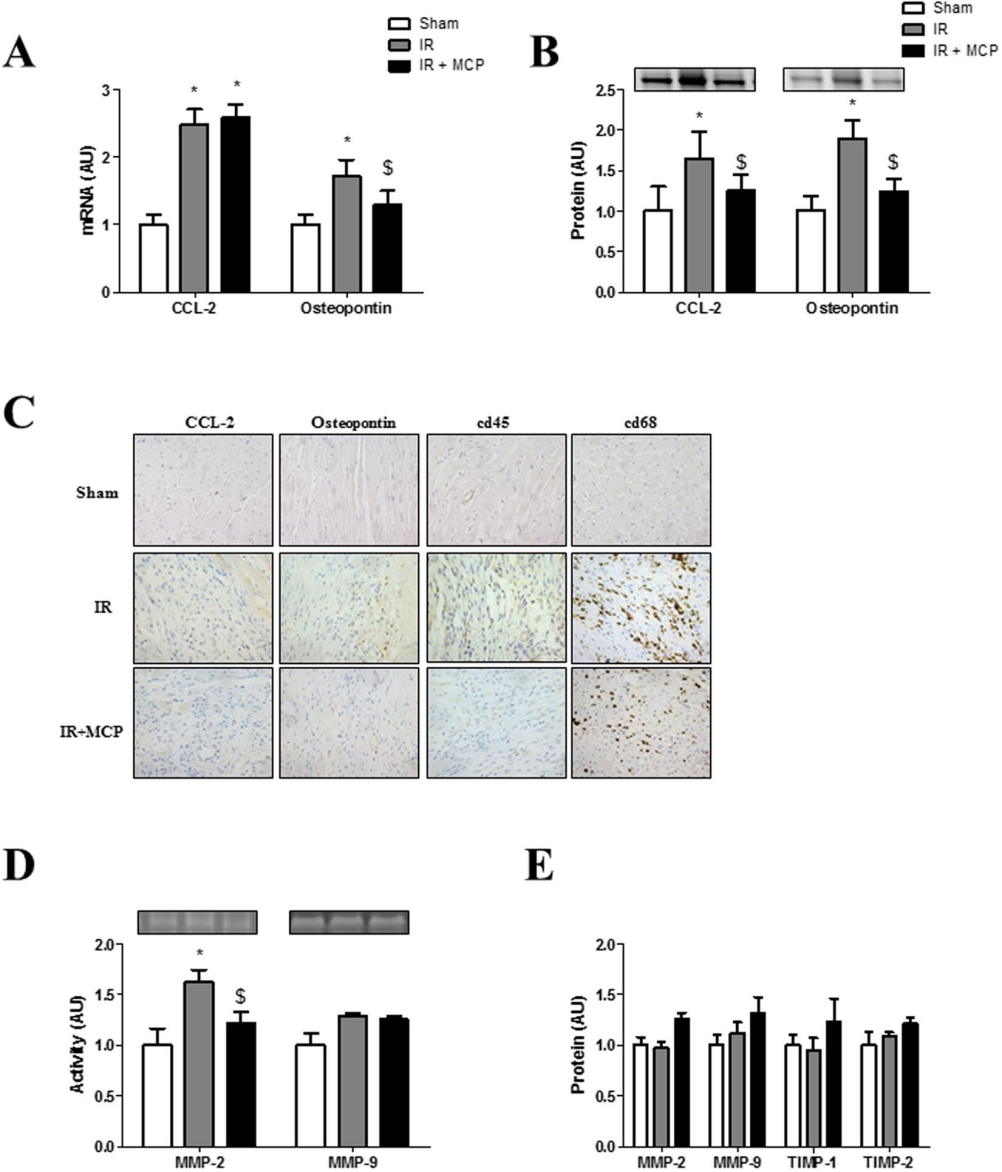
Effects of pharmacological inhibition of Gal-3 on inflammation and cardiac MMPs. (**A**) Quantification of CCL-2 and osteopontin in hearts at mRNA and (**B**) protein levels. (**C**) Representative microphotographs of myocardial sections stained for CCL-2, osteopontin, cd45 and cd68. (**D**) MMP-2 and MMP-9 activities were performed by zymography. (**E**) Quantification of MMP-2, MMP-9, TIMP-1 and TIMP-2 in hearts from rats at protein levels. Histogram bars represent the mean ± SEM of each group of animals (n ≥ 12 per group) in arbitrary units or as a percentage of staining normalized to HPRT, 18 s and β-actin or stain-free gel for cDNA and protein respectively. Magnification 40x. *p < 0.01 *vs*. Sham, $, p < 0.01 *vs*.IR.

Serum inflammatory markers CRP (C-reactive protein) and IL-1β (interleukin 1-β) were enhanced (p < 0.05) in untreated IR rats and reduced by MCP treatment (Table 1). IR rats presented higher (p < 0.01) both mRNA and protein levels of CCL-2 (chemokine ligand 2, also known as monocyte chemoattractant protein-1) and of the pro-inflammatory osteopontin as compared to controls (Fig. 3A,B). While MCP treatment allowed diminishing mRNA level of osteopontin only (Fig. 3A), it induced decreased (p < 0.01) protein expression of both CCL-2 and osteopontin (Fig. 3A,B). In addition, the increase in immunostainings observed of CCL-2, osteopontin, cd45 and cd68 were lowered in IR rats receiving the Gal-3 inhibitor (Fig. 3C).

Because inflammation and cytokines play a critical role in the activation of MMPs (metalloproteinases) involved in ECM turnover, MMP-2 and MMP-9 activity and expression were assessed, showing that MCP treatment allowed reducing (p < 0.01) the increases in MMP-2 activity (p < 0.05) (Fig. 3D). There was no differences in the protein expression of MMP-2, MMP-9, TIMP-1 and TIMP-2 between the groups (Fig. 3E).

MCP treatment did not affect all measured parameters in the absence of IR.

### Effects of Gal-3 blockade on cardiac fibrosis

**Figure 4:**
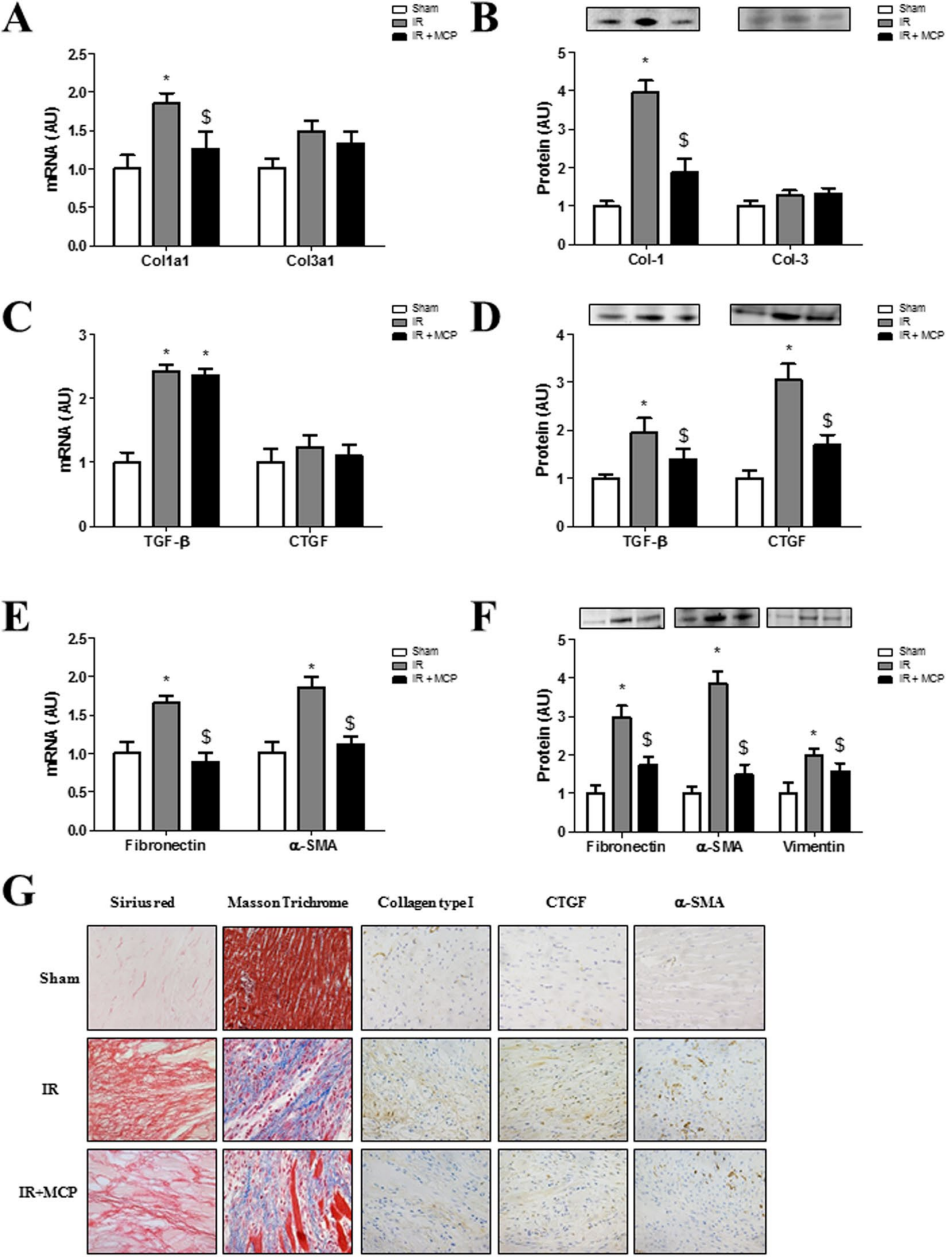
Effects of pharmacological inhibition of Gal-3 on cardiac fibrosis. (**A,B**) Quantification of myocardial collagen type I and type III at mRNA and protein levels. (**C,D**) Quantification of TGF-β and CTGF in hearts from rats at mRNA and protein levels. (**E,F**) Quantification of cardiac fibronectin and α-SMA at mRNA levels and fibronectin, α-SMA and vimentin at protein levels. (**G**) Representative microphotographs of myocardial sections stained for total collagen, Masson Trichrome, collagen type I, CTGF and α-SMA. Histogram bars represent the mean ± SEM of each group of animals (n ≥ 12 per group) in arbitrary units or as a percentage of staining normalized to HPRT, 18 s and β-actin or stain-free gel for cDNA and protein respectively. Magnification 40x. *p < 0.01 *vs*. Sham, $, p < 0.01 *vs*.IR.

LV ischemic zone of rats with IR presented higher (p < 0.01) collagen-1 subunit α1 mRNA expression as well as collagen-1 protein expression, with similar levels of collagen-3 (Fig. 4A,B). Gal-3 blockade decreased collagen-1 expression in the ischemic zone at both mRNA and protein level (p < 0.01) (Fig. 4A,B) without affecting collagen-3. The expression level of the profibrotic mediators TGF-β (Transforming growth factor-beta) and CTGF (Connective-tissue growth factor) were measured. Although TGF-β only displayed increased mRNA level (p < 0.01) in rats with IR (Fig. 4C), MCP treatment allowed preventing (p < 0.01) the increases (p < 0.01) in protein expression of both TGF-β and CTGF (Fig. 4D). Furthermore, in rats with IR, MCP treatment allowed preventing (at least limiting) (p < 0.01) the increases (p < 0.01) in mRNA and protein expressions of the ECM component fibronectin, the α-smooth muscle actin (Figure E and F) and the protein expression of the intermediary filament vimentin (Figure F), all of these proteins being relevant fibroblast activation markers. The reduction in fibrosis and ECM components in the ischemic zone of MCP-treated animals was confirmed after staining of collagens by Sirius red or Masson trichrome, as well as by immunohistochemistry for collagen type I, CTGF and α-SMA (Fig. 4G).

MCP treatment did not affect all measured parameters in the absence of IR.

## Discussion

The purpose of this study was to investigate the effects of Gal-3 inhibition in cardiac alterations associated to IR injury. This study demonstrates that pharmacological Gal-3 blockade induced cardioprotection during IR injury, improving LV perfusion and compliance. The improvement in cardiac function can also be seen in the mitigated extent of cardiac fibrosis and inflammation in the LV of rats treated with the Gal-3 inhibitor MCP. Gal-3 inhibition protects specifically against alterations in ECM without affecting cardiac hypertrophy. Thus, Gal-3 emerges as a new player involved in ECM alterations associated with IR injury.

To explore novel mechanisms by which Gal-3 inhibition affords cardioprotection following acute insults, a model of IR injury was used. Molecular and cellular events underlying IR injury are complex, representing the confluence of divergent biological pathways^13–16^. Recently, it has been reported that Gal-3 can play a protective role on the myocardium following IR injury at 24 h^17^. In Gal-3 knockout mice subjected to IR injury, apoptosis was increased and the antioxidant defenses were decreased as compared to WT mice subjected to IR injury^17^. However, inflammation was decreased in IR Gal-3 knockout mice as compared to IR WT mice^17^. Our results expand these findings showing that 8 days after IR injury, pharmacological Gal-3 blockade improved cardiac inflammation and extracellular matrix remodeling. Interestingly, Gal-3 pharmacological blockade resulted in improving LV perfusion and compliance during recovery compared to IR rats. Although MRI volume measurements did not allow evidencing functional benefits in MCP treated rats as early as 8 days post-IR, invasive hemodynamics clearly evidenced improvement of the diastolic function and MRI assessment indicated improved LV perfusion in comparison to untreated animals. It is likely that early improvement in LV remodeling benefited early improvement in LV function. Moreover, the ischemic zone was lower in rats presenting Gal-3 inhibition. The analysis of the LV remodeling revealed a decrease in inflammation and ECM remodeling, without modifying cardiac hypertrophy. These observations suggest that Gal-3 blockade directly regulates inflammatory cells content and fibroblast function, as it has been previously suggested^17^. Interestingly, these results are in accordance with the fact that Gal-3 is localized at the very sites of fibrosis, co-localizing with macrophages and fibroblasts, but not with cardiomyocytes^18^. In a recent study, we confirmed these data in human myocardium showing that Gal-3 is mainly expressed in ECM and by cardiac fibroblasts. The fact that Gal-3 blockade did not affect cardiomyocyte hypertrophy has been reported in other animal models^5,6,8^. Interestingly, global loss of Gal-3 accelerated cardiac hypertrophy, although the significance of Gal-3 expression in regulation of the biological properties of cardiomyocytes remains unclear^19^. Improvement of cardiac remodeling and decreased LV fibrosis are likely to be linked to the improvement of LV compliance and diastolic function^20–22^. Moreover, heart perfusion occurs during the diastolic phase of the cardiac cycle and therefore can take advantage of improved cardiac compliance. Of note, we have previously shown in rodents that early improvements of cardiac remodeling, diastolic function and myocardial perfusion contribute to mid-long term benefits in slowing down the progression of chronic heart failure post-myocardial infarction^23^.

According to our results, it has been previously shown that Gal-3 is increased at both transcriptional and translational levels in the LV in early ischemic period^17,24^, suggesting a role for Gal-3 in cardiac alterations associated with IR injury. Initially, the acute increase in Gal-3 following IR (24 h) could exert beneficial effects (anti-apoptotic, anti-oxidant)^17^; however, our results show that 8 days after IR Gal-3 promoted deleterious effects. Besides, Gal-3 participates in the secretion of macrophage-related chemokine, pro-inflammatory cytokines and reactive oxygen species production in renal IR injury^25^. Moreover, Gal-3 is expressed by activated microglia/infiltrating macrophages and astrocytes in the ischemic brain and play a role in post-ischemic tissue remodeling by enhancing angiogenesis and neurogenesis^26^.

Previous studies of our group have demonstrated that Gal-3 pharmacological inhibition with MCP prevented cardiac dysfunction, fibrosis and inflammation in several pathophysiological conditions such as hyperaldosteronism^5,8^, obesity, hypertension^6^ or aortic stenosis. Similar beneficial effects of Gal-3 inhibition have been reported on cardiac fibrosis, remodeling and dysfunction in Gal-3 knockout mice subjected to thoracic aortic constriction or angiotensin II treatment^27^. To our knowledge, our study reports for the first time that the pharmacological blockade of Gal-3 is able to prevent cardiac fibrosis, inflammation and functional alterations in an animal model of cardiac IR injury. Thus, these results show the key role of Gal-3 in the early LV remodeling associated with IR injury and the beneficial effects of Gal-3 pharmacological inhibition on cardiac fibrosis and inflammation, the two key processes underlying the cardiac functional alterations which finally affect heart function, leading to HF.

## Materials and Methods

### Myocardial ischemia-reperfusion

Experiments conformed to the 2010/63 directive of the EU and the *Guide for Care and Use of Laboratory Animals* of the US National Institute of Health (No. 85–23). All animal protocols were approved by Haute-Normandie Ethics Board (authorization no.01307.01). After ketamine/xylazine anesthesia (150 and 5 mg.kg^−1^ respectively IP), 12-week old male Wistar (Janvier Labs, Saint Berthevin, France) were subjected to either sham surgery or ischemia-reperfusion (IR) due to transient ischemia provoked by temporary left coronary artery occlusion (45 min) followed by reperfusion, the latter being verified visually before closing the chest, as previously described^28^. Rats received Modified Citrus Pectin (MCP) (EcoNugenics) treatment one day before IR and 8 days following reperfusion at the dose of 100 mg kg^−1^ per day in the drinking water.

### Magnetic Resonance Imaging for myocardial perfusion and LV function

Magnetic resonance imaging (MRI) measurements were performed 8 days after surgery. Myocardial tissue perfusion in the ‘viable’ part of the LV free wall and in the interventricular septum was evaluated in anesthetized rats (sodium methohexital; 50 mg.kg^−1^, IP) using a MRI (Bruker Biospec 4.7 Tesla, France) by Arterial Spin Labeling acquisition sequence, as previously described^29,30^. For LV function, pictures were obtained by retrospective acquisition using ‘intragate’ auto-triggered sequence under Paravision 5.1 (Brucker) that notably allows determining the Heart Rate. Post processing of ‘intragate’ sequence was performed in an average of 9 levels along the LV long-axis, for determining LV tridimensional volume in end-diastole and end-systole, LV Ejection Fraction, Stroke Volume and Cardiac Output (CAAS, pie medical imaging).

### LV Hemodynamics

Rats were anesthetized (sodium methohexital, 60 mg.kg^−1^, IP) and the carotid artery cannulated with a pressure-volume catheter (SPR839, Millar-Instruments, USA) to record arterial pressure (mmHg), after which the catheter was advanced into the LV. Pressure-volume loops were recorded at baseline, and during loading by gently occluding the abdominal aorta with a cotton swab, allowing the calculation with IOX^TM^software (EMKA, France) of dP/dt_max_, dP/dt_min_ max (mmHg/s), LV end-systolic and end-diastolic pressures (mmHg), and LV end-systolic and end-diastolic pressure-volume relations as indicators of load-independent LV passive compliance and contractility respectively.

### Real-time reverse transcription PCR

Total RNA was extracted with Trizol Reagent (Euromedex, Strasbourg, France) and purified using the RNeasy kit, according to the manufacturer’s instructions (Qiagen, Hilden, Alemania). First strand cDNA was synthesized according to the manufacturer’s instructions (Roche, Basilea, Suiza). Quantitative PCR analysis was then performed with SYBR green PCR technology (Bio-Rad, California, USA) (Table S1). Relative quantification was achieved with MyiQ (Bio-Rad, California, USA) software according to the manufacturer’s instructions. Data were normalized by HPRT (Hypoxanthine Guanine Phosphoribosyltransferase) and β-actin levels and expressed as percentage relative to controls. All PCRs were performed at least in triplicate for each experimental condition.

### Western blot analysis

Aliquots of 20 µg of total proteins were prepared from cardiac homogenates, electrophoresed on SDS polyacrylamide gels and transferred to Hybond-c Extra nitrocellulose membranes (Amersham Biosciences, Little Chalfont, UK). Membranes were incubated with primary antibodies for: Gal-3 (Thermo Scientific, Massachusetts, USA; dilution 1/500), collagen type I (Santa Cruz, Texas, USA; dilution 1:500), collagen type III (Santa Cruz, Texas, USA; dilution 1:500), connective tissue growth factor (CTGF; Torrey Pines Biolabs Inc., California, USA; dilution 1:1000), transforming growth factor-beta (TGF-β; Abcam, Cambridge, USA; dilution 1:1000), fibronectin (Santa Cruz, Texas, USA; dilution 1:500), α-smooth muscle actin (α-SMA; Sigma, Missouri, USA; dilution 1:1000), vimentin (Sigma, Missouri, USA; dilution 1:1000), Chemokine Ligand 2 (CCL2; Santa Cruz, Texas, USA; dilution 1/500), Osteopontin (OPN; Santa Cruz, Texas, USA; dilution 1:500), growth differentiation factor (GDF)-15 (Thermo Scientific, Massachusetts, USA; dilution 1:500). After washing, detection was made through incubation with peroxidase-conjugated secondary antibody, and developed using an ECL chemiluminescence kit (Amersham Biosciences, Little Chalfont, UK). After densitometric analyses, optical density values were expressed as arbitrary units. Results are expressed as an n-fold increase over the values of the control group in densitometric arbitrary units. All Western Blots were performed at least in triplicate for each experimental condition.

### ELISA

Brain natriuretic peptide (BNP) was measured in plasma samples by ELISA (Abcam, Cambridge, USA), as well as C-Reactive Protein (CRP), IL-1β, MMP-2, MMP-9, TIMP-1 and TIMP-2 concentrations (R&D Systems, Minnesota, USA) according to the manufacturer’s instructions.

### Gelatin zymography

Aliquots of 20 µg of total proteins were resolved on a 10% SDS polyacrylamide gel containing 0.3% gelatin. The gel was rinsed three times for 15 min with a solution of 2.5% Triton X 100 to remove SDS and renature the proteins, followed by incubation for 48 h at 37 °C in 1000 mmol/l Tris-HCl, pH 7.5 with 1000 mmol/l CaCl_2_ and 5000 mmol/l NaCl to promote degradation of gelatin. Gels were fixed in 40% methanol and 10% acetic acid, and then stained for 30 min in 0.25% Coomassie blue R-250 to identify proteolytic activity of MMPs.

### Histological and immunohistological evaluation

Cardiac tissue samples were dehydrated, embedded in paraffin and cut in 5 μm-thick sections. Sections were stained with Masson trichrome and picrosirius red. The area of the ischemic zone was determined in Masson trichrome sections and it was expressed as a percentage relative to the total LV area. For cardiomyocyte cross sectional area, at least 50 cardiomyocytes per section were measured. The area of interstitial fibrosis was identified as the ratio of interstitial fibrosis to the total tissue area. For each sample, 15 to 20 fields were analyzed with a 40X objective under transmitted light microscope (Leica DM 2000). Quantitative analysis was performed using an analysis system (Leica LAS 4.3). Two independent researchers unaware of the experimental groups performed the analysis.

For immunochemistry, slides were treated with H_2_O_2_ for 10 min to block peroxidase activity. All samples were blocked with 5% normal goat serum in PBS for 1 h and incubated for 1 h with Gal-3 (Thermo Scientific, Massachusetts, USA; dilution 1:50), Collagen type I (Santa Cruz, Texas, USA; dilution 1:50), CTGF (Abcam, Cambridge, USA; dilution 1:50), α-SMA (Sigma, Missouri, USA; dilution 1:100), CCL2 (Santa Cruz; dilution 1:50), OPN (Santa Cruz, Texas, USA; dilution 1:50), cd45 (Santa Cruz, Texas, USA; dilution 1:50), cd68 (Abcam, Cambridge, USA; dilution 1:50), and MMP-2 (Thermo Scientific, Massachusetts, USA; dilution 1:100), washed three times, and then incubated for 30 min with the horseradish peroxidase-labeled polymer conjugated to secondary antibodies (Dako Cytomation, Snata Clara, USA). The signal was revealed by using DAB Substrate Kit (BD Pharmingen, San Jose, USA). As negative controls, samples followed the same procedure described above but in the absence of primary antibodies.

### Statistical analyses

For animal studies, data are expressed as mean ± SEM. Normality of distributions was verified by means of the Kolmogorov–Smirnov test. Data were analyzed using a one-way analysis of variance, followed by a Newman-Keuls to assess specific differences among groups or conditions. Functional cardiac parameters were compared using one-way ANOVA followed, in case of significance, by Tukey test for multiple comparisons. Statistics were performed using GraphPad Software Inc. The predetermined significance level was P < 0.05.

### Statement of Ethics

Animal experiments conform to internationally accepted standards and have been approved by the appropriate institutional review body.

## Supplementary information

Supplementary material

## Data Availability

Data available on request from the authors.
