# Supplementary material for: Myocardial Injury After Ischemia/Reperfusion Is Attenuated By Pharmacological Galectin-3 Inhibition
Source: Sci Rep. 2019 Jul 3;9:9607. doi: 10.1038/s41598-019-46119-6 (PMC6610618; doi:10.1038/s41598-019-46119-6)
Supplement: Supplementary file 1 — Supplementary material [file 41598_2019_46119_MOESM1_ESM.pdf]

MYOCARDIAL INJURY AFTER ISCHEMIA/REPERFUSION IS ATTENUATED BY PHARMACOLOGICAL GALECTIN-3 INHIBITION

Jaime Ibarrola[1], Lara Matilla[1], Ernesto Martinez-Martinez[1], Alexandre Gueret[2], Amaya Fernández-Celis[1], Jean-Paul Henry[2.3], Lionel Nicol[2,3], Frederic Jaisser[4,5], Paul Mulder[2,3], Antoine Ouvrard-Pascaud†[2,3], Natalia López-Andrés†*[1,5]

† These authors contributed equally to this work.

[1] Cardiovascular Translational Research. Navarrabiomed, Complejo Hospitalario de Navarra (CHN), Universidad Pública de Navarra (UPNA), IdiSNA. Pamplona. Spain; [2] Normandie University UNIROUEN, [3] INSERM UMR1096, Rouen, France ; [4] Inserm 1138, Institut des Cordeliers, Paris, France. [5] INSERM Institut National de la Santé et de la Recherche Médicale, Centre d’Investigations Cliniques-Plurithématique 1433, UMR 1116 Université de Lorraine, CHRU de Nancy, France

SUPPLEMENTARY MATERIAL

Table S1: Primers used in rats in real time PCR analysis

**Gene**	**Primer**	**Sequence (5´to 3´)**
**α-SMA**	Forward	GAA GGA ATA GCC ACG CTC AG
	Reverse	TGT GCT GGA CTC TGG AGA TG
**β-actin**	Forward	CCT CTA TGC CAA CAC AGT GCT GTC T
	Reverse	GCT CAG GAG GAG CAA TGA TCT TGA
**Col 1a1**	Forward	GCC TCC CAG AAC ATC ACC TA
	Reverse	ATG TCT GTC TTG CCC CAA GT
**Col 3a1**	Forward	CTCACCCTGCTTCACCCCTCTC
	Reverse	TGGACATATTGCACAACATTCTC
**TFG-β**	Forward	CAG AAG TTG GCA TGG TAG CC
	Reverse	TGC TTC AGC TCC ACA GAG AA
**CTGF**	Forward	GAG TCG TCT CTG CAT GGT CA
	Reverse	CCA CAG AAC TTA GCC CGG TA
**HPRT**	Forward	AGG ACC TCT CGA AGT GT
	Reverse	ATTCAAATCCCTGAAGTACTCAT
**CCL2**	Forward	TTC CTT ATT GGG GTC AGC AC
	Reverse	CAG TTA ATG CCC CAC TCA C
**OPN**	Forward	ATG AGA CTG GCA GTG GTT
	Reverse	GCT TTC ATT GGA GTT GCT
**Fibronectin**	Forward	GGG GTC ACG TAC CTC TTC AA
	Reverse	TGG AGG TTA GTG GGA GCA TC
